# Assisted peritoneal dialysis compared to in-centre hemodialysis – an observational study of outcomes from the Swedish Renal Registry

**DOI:** 10.1186/s12882-024-03799-1

**Published:** 2024-10-14

**Authors:** Helena Rydell, Mårten Segelmark, Naomi Clyne

**Affiliations:** 1 Department of Clinical Science, Intervention and Technology (CLINTEC), Karolinska Institutet, Solna, Sweden; 2 Department of Renal Medicine, Karolinska University Hospital Huddinge, , M99, Stockholm, 141 86 Sweden; 3 Swedish Renal Register, Jönköping, Sweden; 4 Department of Clinical Sciences in Lund, Department of Endocrinology, Nephrology and Rheumatology, Lund Universityand, Skane University Hospital , Lund, Sweden

**Keywords:** Assisted peritoneal dialysis, In-center hemodialysis, Hospitalization, Dialysis modality discontinuation

## Abstract

**Background:**

In-center hemodialysis (IHD) is the most common dialysis modality. Assisted peritoneal dialysis (assPD) is an option for frail and/or incapacitated patients. Both modalities can be used to alleviate uremic symptoms towards the end of life. There are few studies comparing these modalities. The primary aim is to compare hospital admissions between assPD and IHD. The secondary aim is to compare continuation of the dialysis modality and patient survival.

**Methods:**

Patients > 65 years, registered in the Swedish Renal Registry (SRR) and starting dialysis 2010–2017 were eligible for inclusion. Patients starting on assPD were matched with patients starting on IHD according to sex, Charlson Index, age and date for start of dialysis. Data were collected from SRR and other registries.

**Results:**

During the first year, patients on assPD and IHD had in median one (IQR 0–5.0; 0–4.0) hospitalization (*p* = 0.412). There was no significant difference after two years, in the annual number of days admitted to hospital, in hospitalizations with cardiovascular or infectious disease diagnoses or continuation of the dialysis modality, respectively. However, patients on assPD had a worse median survival (1.1 years IQR 0.6–2.1; IHD 3.1 years IQR 0.2–5.8; *p* < 0.001).

**Conclusion:**

In this study patients starting assPD, often as a palliative treatment, showed no difference compared to IHD concerning the number of hospitalizations, number of days in hospital/year or continuation of the dialysis modality. Patients on assPD had a worse survival, which is likely due to residual confounding. Without that, patients on assPD would probably have lower number of hospitalizations. Despite limitations due to the retrospective observational design of the study, the results indicate that assPD is a feasible alternative to IHD when self-care dialysis is not possible and/or IHD too arduous.

**Supplementary Information:**

The online version contains supplementary material available at 10.1186/s12882-024-03799-1.

## Background

The number of patients with kidney replacement therapy (KRT) is increasing worldwide (https://usrds-adr.niddk.nih.gov/2022). In most European countries the incidence of KRT is highest in individuals aged ≥ 75 years (https://usrds-adr.niddk.nih.gov/2022) [1]. Even though the proportion of patients with a kidney transplant is increasing in many countries, most elderly patients are treated with hemodialysis (HD) (https://usrds-adr.niddk.nih.gov/2022) [2]. For the elderly or frail person, hemodialysis has some disadvantages, exhausting transfers several times per week to the dialysis unit, and postdialysis fatigue. Previous studies have shown an increased risk of dementia [3] and hemorrhagic stroke [4] in patients on HD compared with peritoneal dialysis (PD). Furthermore, self-care PD is suitable for many patients but not for all. Assisted PD (assPD) is a way of enabling PD for frail or older patients with a high burden of comorbidity, who cannot perform PD independently. However, assPD is still not available in all countries and Nephrology units [5]. In Sweden assPD has been available since the 1990ies [6], yet to date only 4% of all patients on dialysis in Sweden are treated with assPD and the modality is still not available in all regions [2]. The main provider responsible for the staff costs for assistance during PD varies in the Swedish health care regions, in some regional health care is responsible while in others municipal elderly care provides this service. Patients in all regions are followed by nephrologists who are responsible for dialysis prescription and medications.

There are few earlier studies comparing assPD and in-center hemodialysis (IHD), the dialysis modalities suitable for frail, incapacitated and/or elderly patients. Oliver et al. showed that patients on assPD or IHD had the same frequency of hospitalizations [7]. Iasere et al. showed a similar quality of life and physical function for patients on assPD and IHD, but a higher satisfaction with the dialysis modality for patients with assPD [8, 9]. There are some studies comparing assPD and self-care PD, but for the oldest, frailest and/or incapacitated patients with multiple comorbidities self-care PD is not a clinically relevant option [10–13].

The total Swedish population of patients on assPD are included in the Swedish Renal Registry (SRR), a high-quality national registry with all Swedish Nephrology units connected to the registry [2]. Patients at all stages of CKD and all kidney replacement therapies are included. The coverage is almost 100% for patients on maintenance dialysis [14] and about 93–98% of the patients on dialysis are included in the yearly cross-sectional dialysis surveys with granular data on dialysis modality, prescriptions and laboratory parameters [2].

Sweden has a high proportion of patients on PD compared to many other countries, 20–25% (https://usrds-adr.niddk.nih.gov/2022), (https://www.medscinet.net/snr/). In Sweden assPD is usually prescribed as an active, palliative treatment for a selected group of patients, for whom in-center hemodialysis (IHD) could be a possibility, but is not deemed the preferable choice, or for whom comprehensive conservative treatment might be an alternative option. Under these circumstances it is of interest to investigate how often these patients are hospitalized and how often the dialysis modality is discontinued.

The primary aim of the present study is to compare the utilization of inpatient care, i.e. hospital admissions and days in hospital, for patients with assPD or IHD as initial KRT. The secondary aim is to compare continuation of dialysis modality and patient survival between assPD and IHD.

## Methods

### Inclusion criteria and definitions of groups

All patients, 65 years or older, registered in the SRR and starting dialysis between 2010 and 2017 were eligible for inclusion if they fulfilled the inclusion criteria of assPD as initial KRT. In the SRR, the exact dates of start of KRT and switches between IHD, HHD, PD and kidney transplantation are registered. In addition, there is a yearly cross-sectional survey of all patients on dialysis (CSS) in Sweden. The distinction between self-administered and assPD can only be found in the CSS. The exact starting dates of assPD were accessed from patients’ files. There is no information in the SRR on whom provides assistance, it could be either health care staff or relatives.

To be eligible for the present study a patient should have started PD or IHD as their first dialysis modality, which should have been registered in the first CSS as either assPD or IHD. Among patients registered as having assPD in the first CSS, those with IHD or self-care PD for more than 1 month before start of assPD were excluded. Among patients registered as having IHD, those with PD before start of IHD were not eligible for matching. A recovered kidney function, start of home hemodialysis, a kidney transplantation or a registration on the kidney transplant waiting list before or after start of dialysis were exclusion criteria for both groups, as all these events would most likely be more common among patients on IHD. Patients on IHD should have been registered in the SRR’s section for chronic kidney disease not on KRT (SRR-CKD) > 1 month before start of dialysis, thus excluding patients starting on IHD because of acute kidney failure.

### Matching

Patients on assPD were matched 1:1 with patients on IHD according to sex, exact Charlson Comorbidity Index, age (+ / − 3 years) and date for start of KRT (+ / − 3 years). Matching was performed in loops with the best possible matching patients on IHD for each patient on assPD until there was no available matches for all patients on ass PD. Charlson Comorbidity Index [15] was defined without age and by using all discharge diagnoses in the Swedish Inpatient Registry until the start date of KRT as previously described [16]. Kidney failure was not included in the index as previously suggested for populations on maintenance dialysis [17]. Matching was performed at day 0 of RRT.

#### Collection of data

Dates of start and changes in KRT, dates of birth and kidney diagnosis were collected from the SRR. Laboratory values and dialysis prescriptions were collected from the first CSS in the SRR. Discharge diagnoses and dates of hospital admissions were collected from the Swedish Inpatient Registry, dates of death from the Swedish Mortality Database and information about registrations on waiting-lists for transplantation were collected from Scandiatransplant. Data from the SRR and patients’ files were individually linked to the other registries based on personal identification numbers and were then anonymized.

#### Ethical approval

The study was approved by the Swedish Ethical Review Authority (2017/760 and 2019–02810).

#### Follow up

As discrimination between self-administered and assPD is not registered at start, but only in the yearly CSS in the SRR, patients starting on assPD who had changed dialysis modality or died before the first CSS could not be identified in the registry. Therefore, only patients on assPD or IHD who had survived until their first CSS were included in the study. Day 0 in the study was defined as the day of the first CSS. All patients were followed until death or September 1st 2019. Results are given for one year and two years, respectively, of follow up after the first CSS.

### Comparison of hospital admissions

Time to first all-cause admission after the first CSS was compared as well as annual all-cause hospital admission rates and annual days admitted during one and two years after the first CSS. Time to first all-cause admission was performed as an intention-to-treat analysis, not considering changes to other modalities, censoring was performed only at the dates of death in this analysis. The admission rates and annual days admitted were calculated as the number of admissions during the exact days of follow up for each patient, maximally one or two years. The only reason for shorter follow up was death as the analysis was performed as intention-to-treat. Acute and elective admissions were included in the analysis. There is no separation between these causes in the Swedish Inpatient Registry.

Separate comparisons were performed for annual hospital admission rates and number of days admitted with cardiovascular- or acute infectious disease diagnoses. Diagnoses used for defining an admission as due to cardiovascular or infectious disease according to ICD 9 and 10, have been described previously [16].

### Patient survival and continuation of the dialysis modality

Survival was calculated from the date of the first CSS. The survival analysis was performed as an intention-to-treat analysis (ITT), patients were considered at risk also after switching to other modalities of KRT. Continuation of dialysis modality was compared between assPD and IHD and defined as a switch to another dialysis modality after the first CCS. In this analysis, censoring was performed at death and the end of the study. There is no registration of discontinuation of any form of dialysis and end-of-life care in the SRR.

### Statistical analysis

All statistical analyses were performed using IBM SPSS Version 23. The follow up started at the first CSS, less than 1 year after start of RRT. Wilcoxon signed rank test was used for comparisons of frequency of hospital admissions and annual days admitted. Poisson regression was used for comparisons of all-cause annual days admitted with adjustment for kidney diagnosis and days admitted to hospital during the last year before start of dialysis. Kaplan–Meier estimate and Breslow test were used for comparisons of time to change of dialysis modality, time to first hospital admission and death. Cox regression was used in comparisons of patient survival with adjustment for factors that was not used in the matching, days admitted to hospital one year before start and kidney diagnoses. A supplementary analysis was performed for patient survival with additional adjustment for additional factors; laboratory values at the first CSS and vintage at start. Results are given as medians and interquartile ranges (IQR). P-value < 0.05 was considered as statistically significant.

## Results

### Patient characteristics and laboratory values

Among patients starting dialysis 2010–2017, 207 patients had assPD at their first cross-sectional survey. Of these, 118 patients fulfilled all study criteria and could be matched with 118 patients with IHD (Suppl Fig. 1). The median age was 79 years in both groups and 64% were male. Only 8% in both groups had a Charlson Comorbidity Index of 0, while the proportions of patients with an index of 1, 2 or 3 were 30%, 31% and 31%, respectively. For most separate diagnoses included in the comorbidity index there were only small differences between the groups but cerebrovascular disease was more common among patients on assPD and congestive heart failure was more common among patients on IHD (Suppl Table 1). The cohorts were not matched with respect to kidney disease but the most common kidney diagnosis in both groups was diabetic nephropathy, followed by hypertension. Patients starting assPD had more days in hospital during the last year before start of dialysis compared with patients starting IHD, in median 27 days compared with 16. The median dialysis vintage at the first CSS was equal between the groups, 0.5 years (Table 1). Patients on ass PD had lower median plasma albumin (28 g/L) and higher CRP (22 mg/L) compared with patients on IHD (33 g/L; 8 mg/L; Table 2). There were missing data for total Kt/V urea for patients on assPD but not for other variables.

**Table 1:** Characteristics at start of dialysis for patients with assPD or IHD as initial KRT

	**AssPD**	**IHD**
Total number *(n)*	118	118
Age at start *median (IQR) years*	79 (74–82)	79 (74–83)
Sex *male percent (n)*	64% (76)	64% (76)
Charlson comorbidity index at start *percent (n)*		
0	8% (10)	8% (10)
1	30% (35)	30% (35)
2	31% (36)	31% (36)
3	31% (37)	31% (37)
Kidney diagnosis *percent (n)*		
APCKD	4% (5)	5% (6)
Diabetic nephropathy	31% (38)	31% (36)
Hypertension	29% (36)	26% (31)
Renovascular disease	0% (0)	1% (1)
Glomerulonephritis	2% (3)	6%( 7)
Pyelonephritis	4% (5)	3% (4)
Other	12% (16)	17% (20)
Unknown	12% (15)	11% (13)
Vintage at CSS *median years*	0.5 (0.2–0.7)	0.5 (0.2–0.8)
Days admitted one year before start *median (IQR) n*	27 (10–46)	16 (7–42)

*AssPD* Assisted peritoneal dialysis, *IHD* in-center hemodialysis, *KRT* Kidney replacement therapy, *IQR* Interquartile range, *APCKD* Adult polycystic kidney disease, *CSS* Cross sectional survey

**Table 2:** Laboratory values at first CSS for patients with assPD or IHD as initial KRT

	**AssPD**	**IHD**
Total number *(n)*	118	118
Albumin g/L *median (IQR;n)*	28 (25–32; 117)	33 (31–36; 98)
CRP mg/L *median (IQR;n)*	22 (6–61; 106)	8 (5–27; 93)
Phosphate mmol/L *median (IQR;n)*	1.4 (1.1–1.7; 111)	1.4 (1.2–1.8; 93)
Hemoglobin g/L *median (IQR;n)*	116 (107–127; 113)	112 (102–123; 93)

*CSS* Cross sectional survey, *AssPD* Assisted peritoneal dialysis, *IHD* In-center hemodialysis, *KRT* Kidney replacement therapy, *IQR* Interquartile range, *CRP* C-reactive protein

### Dialysis prescriptions and doses

Most patients with assPD, 89% (105), were treated with CAPD at the first CSS. Patients on CAPD had in median 4 (IQR 3–4) changes per day with a median total volume of 8 (IQR 6–8) liters at their first CSS. Only 3 patients had less than 3 daily changes. For patients on APD the median daily volume was 11.0 (IQR 10.0–13.5) liters. Total kt/V urea is not a mandatory variable in the registry and the completeness is low for all patients on PD [2]. Only 50 patients in the study cohort had a registered total Kt/V urea at their first CSS. Among these 39 (78%) had a total weekly Kt/V urea > 1.7. Three patients were anuric.

No patient on IHD had HDF. Most patients had an AV-fistula 47% (55), followed by a tunneled central dialysis catheter 42% (49). The majority was prescribed 3 dialysis sessions weekly, 71% (84), and 25% (30) had incremental dialysis, twice weekly. Only 4 patients had a higher dialysis frequency. The tot SAN Kt/V was > 2.1 for 57% (67) of the patients on IHD.

### Changes and continuation of the dialysis modalities

Among patients with assPD, 5 had a short period, less than 1 month (according to the inclusion criteria) on IHD before start of assPD. After a period on assPD, 11 changed to IHD, in median 1.2 years (IQR 0.5–1.8) after their first cross-sectional survey and 1.7 years (IQR 1.0–2.1) after start of dialysis. Among patients with IHD, four changed to PD, in median 1.6 years (IQR 0.2–0.7) after their first CSS and 2.0 years (IQR 0.4–0.9) after start of dialysis. No patient was lost to follow up.

**Fig. 1:**
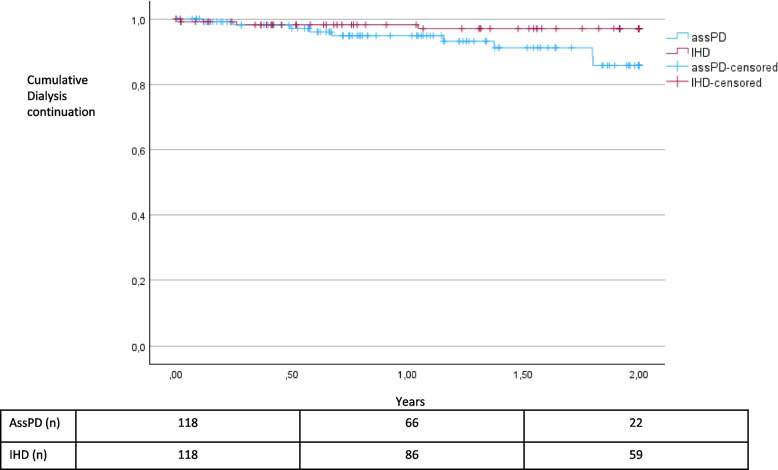
Continuation of the dialysis modality after the first CSS for matched patients with assPD and IHD (with changes in dialysis modalities considered as events and with censoring at the dates of death; *p* = 0.052)

There was no significant difference in continuation of dialysis modality after the first CSS between assPD and IHD (*p* = 0.052). The one- and two-years’ continuation rate was 95% and 86%, respectively, for assPD and 98% and 97% for IHD (Fig. 1).

### Comparison of hospital admissions

Among patients with assPD, 47% had no admissions during the first year of follow up and 41% during the two years of follow up. Among patients with IHD 43% and 39% had no admissions during the same time periods. The median time to first all-cause hospital admission was 0.7 (IQR 0.2-non applicable) years for patients on assPD and 0.4 (IQR 0.1-; *p* = 0.226) for patients on IHD. The median annual all-cause hospitalization rate during the first year of follow-up was 1 both for patients on assPD and IHD (*p* = 0.412; Table 3). During the two years of follow-up, the annual all-cause hospitalization rate was 1,5 for patients on assPD and 1 for patients on IHD but the difference was not statistically significant (*p* = 0.077).

**Table 3:** Annual hospitalizations for patients with assPD or IHD as initial KRT

	**All-cause**				**Infectious disease**				**Cardiovascular disease**			
	One year after CSS		Two years after CSS		One year after CSS		Two years after CSS		One year after CSS		Two years after CSS	
	n	Days n	n	Days n	n	Days n	n	Days n	n	Days n	n	Days n
AssPD *Median (IQR)*	1.0 (0–5.0)	1,0 (0–32.3)	1.5 (0–4.8)	5.2 (0–33.3)	0 (0–1.0)	0 (0–2.5)	0 (0–1.5)	0 (0–9.4)	0 (0–0)	0 (0–0)	0 (0–0.5)	0 (0–0)
IHD *Median IQR*	1.0 (0–4.0)	2.0 (0–20.0)	1.0 (0–3.2)	3.1 (0–14.6)	0 (0–1.0)	0 (0–3.3)	0 (0–0.5)	0 (0–3.6)	0 (0–0)	0 (0–0)	0 (0–0.5)	0 (0–1.5)
*P* value	0.412	0.138	0.077	0.009	0.349	0.502	0.05	0.079	0.667	0.935	0.987	0.731

*AssPD* assisted peritoneal dialysis, *IHD* in-center hemodialysis, *KRT* Kidney replacement therapy, *CSS* Cross sectional survey, *IQR* interquartile range

There was no significant difference in the median all-cause number of days admitted to hospital during the first year of follow-up between patients on assPD and IHD (1.0 and 2.0; *p* = 0.138). During the two years of follow-up, patients on assPD had a higher number of days admitted compared with patients on IHD, 5.2 and 3.1, respectively (*p* = 0.009). After adjustment for kidney diagnosis and the number of days admitted during the last year before start of dialysis there was no statistically significant difference between patients on assPD and IHD (*p* = 0.079).

Most patients in both groups had no hospital admission with an infectious disease diagnosis or cardiovascular diagnosis during the first year of follow-up or during the two years of follow-up, there were no significant differences between the groups (*p* > 0.05; Table 3). Among patients on assPD, 17 had in total 53 hospitalizations with peritonitis during the two years of follow-up (1–8 per patient).

### Patient survival

**Fig. 2:**
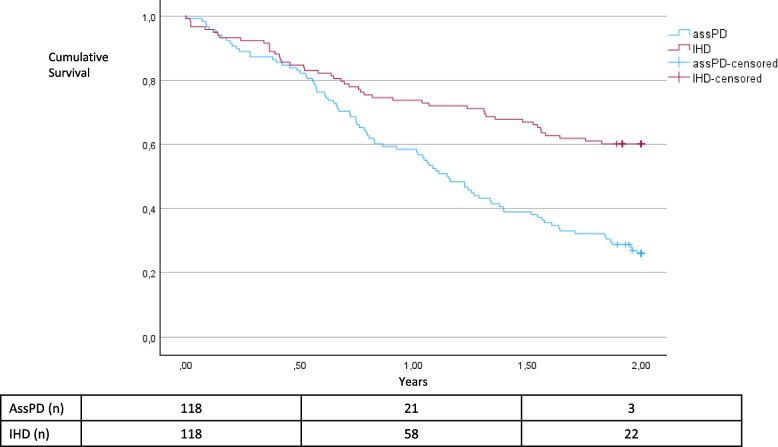
Patient survival from the first CSS for matched patients with assPD and IHD (IHD > assPD, *p* < 0.001). The analysis was performed as intention-to-treat, with censoring performed only at the end of study

Median patient survival was significantly shorter for patients with assPD compared with IHD, 1.1 years (IQR 0.6–2.1) compared with 3.1 years (IQR 0.2–5.8; *p* < 0.001). Patient survival after the first CSS was 59% after 1 year and 26% after 2 years for patients on assPD; survival was 74% after 1 year and 60% after 2 years for patients on IHD (Fig. 2). Patient survival remained lower for patients on ass PD compared with IHD after adjustment for days in hospital one year before start and kidney diagnosis (HR 2.504; *p* < 0.001). A supplementary analysis with all these factors as well as adding plasma albumin, CRP and hemoglobin at the first CSS and dialysis vintage at start (HR 2.917; *p* < 0.001) did not change the difference in survival between assPD and IHD.

## Discussion

This registry-based, nation-wide study showed that patients with assPD as initial KRT had a similar frequency of hospital admissions and number of days per year in hospital compared with patients with IHD as initial KRT. There was no difference in discontinuation of dialysis modality between the groups. Moreover, only 11 out of 118 patients starting on assPD changed dialysis modality and among patients alive after 2 years, 89% were on assPD. Patient survival was significantly lower for patients on assPD compared with IHD after 1 and 2 years on the respective treatment modality.

The hospitalization rates, one admission per year during the first year, for patients with assPD and matched patients with IHD, were similar compared with previous studies by Oliver et al. [7, 18] However, the number of days/year admitted to hospital for patients on assPD, 1.0 during year one and 5.2 during the two years following the CSS, were lower compared with all previous studies, which have reported 11–53 days per year in hospital for patients on assPD [7, 18–20]. There are several factors which might impact these differences. Other studies analyzed data from earlier time periods, 1998–2013, and the overall prognosis has improved for patients on dialysis during the last decades. There are also differences in the organization of healthcare and in the number of hospital beds in different countries. The number of hospital beds in Sweden has decreased since the nineties to a low level compared with other high-income countries, although concomitantly nursing care has shifted from in-hospital to being administered in the home [2, 21].

There was no difference between assPD and IHD for hospitalizations with cardiovascular diagnoses and a nonsignificant trend towards a higher number of admissions with infectious disease diagnoses for patients with assPD during the two years following the first CSS. In accordance, Oliver et al. did not find a significantly increased overall risk of hospitalizations with infectious disease diagnoses in patients on assPD. On the other hand, they reported a significantly increased number of days per year in hospital for peritonitis in patients on assPD compared with days in hospital for HD catheter-related bacteremia for patients on HD [7]. The number of hospitalizations and days admitted could probably be further reduced for patients on assPD if preventive measures against peritonitis were increased and the organization of outpatient care for peritonitis was improved.

Previous studies comparing self-care PD and HD have reported a higher discontinuation rate with PD [22]. In the present study there was a nonsignificant trend towards a higher discontinuation rate with assPD. IHD is often a subsequent alternative modality to both self-care and assPD, when there are medical complications and/or contraindications to PD, such as frequent peritonitis. Oliver et al. also reported a higher proportion of patients changing from assPD to IHD compared with patients on IHD changing to assPD [7]. It is notable, that the continuation rate one year after the first CSS was high in the present study, 95%, for patients on assPD. Previous studies have reported lower one year dialysis modality continuation rates of 58%, 81% and 88% [18, 19, 23]. This could be related to differences in methods between the studies. As we did not have data separating assPD and self-care PD at start of dialysis, all patients starting with assPD and then changing to other modalities prior to or not surviving until their first CSS were excluded from the analysis.

Contrary to the study by Oliver et al., we found a significantly lower survival for patients on assPD compared with IHD [7]. Most previous studies comparing PD, without differentiating between assPD and self-care PD, and IHD have reported similar patient survival rates between the modalities during the first years [24]. Even if we do not have complete data on dialysis effectiveness, there is no evidence supporting that the higher mortality rate was related to an inadequate dialysis dose, as most of the patients had CAPD with four changes per day with a total daily dialysate volume of 8 L. It is more likely that the difference in survival between the dialysis modalities in our study are related to patient selection and residual confounding. In Sweden, assPD is often used as a palliative treatment for patients with a short expected life-span, especially in those with cognitive impairment, advanced cardiac failure or who are deemed too frail to be able to tolerate IHD. Patients on assPD had a higher number of days admitted to hospital the year before starting dialysis compared with patients on IHD. They also had higher CRP and lower plasma albumin levels at their first CSS, which are indicative of malnutrition, and inflammation. Both low plasma albumin and increased CRP are strong indicators of cardiovascular disease and increased mortality in patients on dialysis [25]. Results from the peridialysis study, corroborate that patients starting assPD are a vulnerable group per se. In that study, patients with mental contraindications to self-care PD, had a worse survival compared with patients who started PD or HD as a free choice [26]. If there is residual confounding associated with patients being frailer when they start assPD, the findings in this study support the use of assPD, even stronger, as hospitalizations were still not more frequent for patients on assPD compared with IHD.

The one-year survival in our study was lower, 59% and 74% for patients on assPD and IHD, respectively, compared with 89% for both groups in a previous study by Oliver [7]. The patients in our study had in general poorer health, Oliver et al. did not exclude patients who received a kidney transplant, and over 60% of the patients in our study had a Charlson comorbidity score of 2 or 3. Two earlier studies on survival in patients on assPD also reported a divergent one year survival, 83% [19] and 58% [27] compared to the study by Oliver and our study. The patients assigned to assPD in these studies are a heterogenous group, which makes it difficult to compare survival rates. The survival rate for patients on assPD and IHD, respectively, in our study would probably have been even lower had we not excluded patients who did not survive their first CSS.

Two previous studies have analyzed cost differences between assPD, performed as APD, and IHD. Olsen et al. reported similar costs for patients on assPD and IHD in Denmark [28] and Bevilacqua et al. reported lower costs for assPD in Canada [23] compared with IHD. None of these studies included costs for hospitalizations in their analyses, but neither our study nor the study by Oliver et al. found any differences in hospitalizations. Unfortunately, there is no published analysis on cost differences between these modalities in Sweden.

We acknowledge some limiting factors in our study. Unfortunately, no discrimination between assPD and self-care PD is registered at the start of dialysis in the SRR. Consequently, in this retrospective study, we were unable to classify the groups according to dialysis modality at day 1 on KRT. However, assPD and self-care PD are registered at the annual CSS, which is what we used in this study. There is also a large risk of residual confounding between groups in an observational study. Without residual confounding, the survival would probably have been higher and the hospitalization rates have been lower for patients on assPD. We have no data on frailty, whether patients were living in care homes, their physical function or their capacity for self-care dialysis, severity of cardiac failure or cognitive function, factors that probably differ between groups. Although we have data on dialysis volumes for patients on PD, the estimated Kt/V is only known for a minority of patients. Moreover, we have no information on the frequency of discontinuation of dialysis for palliation or causes of death. The study also has some strong merits. It is one of the largest studies in the field, one of very few comparing assPD and IHD in matched patients. The study population is nationwide and the data is collected from high-quality national registries with a high coverage.

## Conclusions

In conclusion, this study shows that assPD is a good alternative to IHD for frail, elderly or incapacitated patients with a low discontinuation rate of dialysis modality and a similar incidence of hospitalizations and number of days spent in hospital. Thus, enabling them to spend their time at home rather than in a hospital. The survival rate was significantly lower in patients on assPD compared with IHD, most likely due to residual confounding caused by more vulnerable patients on assPD. Without that, patients on assPD would probably have lower number of hospitalizations. Although this retrospective observational registry study was not tailored to elucidate the causes. Due to organizational problems, assPD is not available in all countries and health care regions, thus all patients do not have the possibility to make a person-centered, individualized choice of kidney replacement therapy.

## Supplementary Information

Supplementary Material 1. Figure S1. Inclusion and exclusion of matched patients with assPD and IHD as initial kidney replacement therapy.

Supplementary Material 2. Table S1. Separate comorbidities included in Charlson comorbidity index at start of dialysis for patients with assPD or IHD as initial KRT.

## Data Availability

The data underlying this article will be shared on reasonable request by the corresponding author.

## Abbreviations

AssPD: Assisted peritoneal dialysis
IHD: In-center hemodialysis
SRR: Swedish Renal Registry
