# Supplementary material for: Assisted peritoneal dialysis compared to in-centre hemodialysis – an observational study of outcomes from the Swedish Renal Registry
Source: BMC Nephrol. 2024 Oct 14;25:349. doi: 10.1186/s12882-024-03799-1 (PMC11475596; doi:10.1186/s12882-024-03799-1)
Supplement: Supplementary file 1 — Supplementary Material 1. Figure S1. Inclusion and exclusion of matched patients with assPD and IHD as initial kidney replacement therapy. [file 12882_2024_3799_MOESM1_ESM.docx]

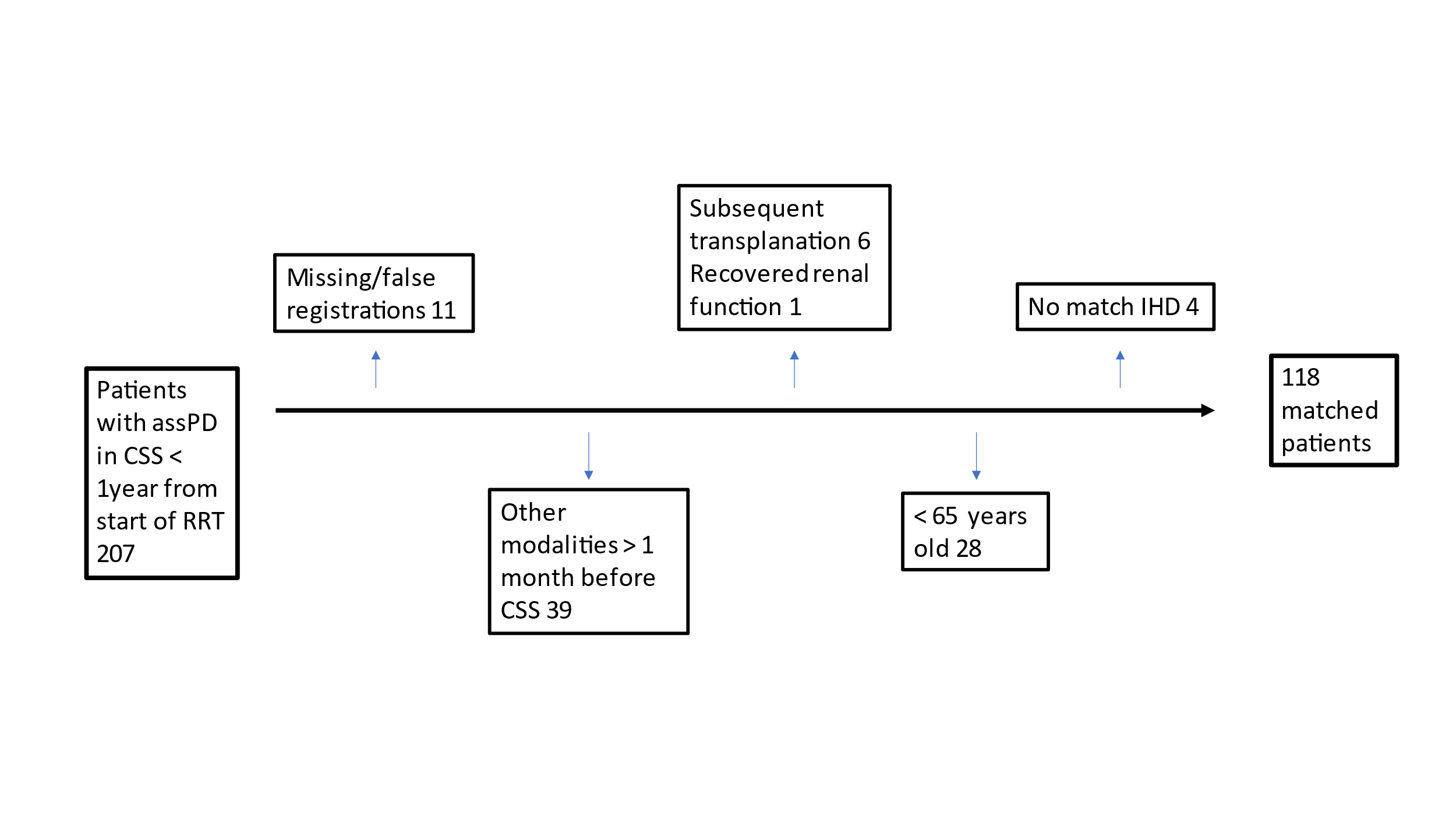


Supplementary figure 1

Inclusion and exclusion of matched patients with assPD and IHD as initial kidney replacement therapy
