# Supplementary material for: Assisted peritoneal dialysis compared to in-centre hemodialysis – an observational study of outcomes from the Swedish Renal Registry
Source: BMC Nephrol. 2024 Oct 14;25:349. doi: 10.1186/s12882-024-03799-1 (PMC11475596; doi:10.1186/s12882-024-03799-1)
Supplement: Supplementary file 2 — Supplementary Material 2. Table S1. Separate comorbidities included in Charlson comorbidity index at start of dialysis for patients with assPD or IHD as initial KRT. [file 12882_2024_3799_MOESM2_ESM.pdf]

Supplementary table 1

Separate comorbidities included in Charlson comorbidity index at start of dialysis for patients with assPD or IHD as initial KRT.

**Comorbidity** *percent (n)*	**AssPD**	**IHD**
Coronary disease	43 % (51)	42 % (49)
Congestive heart failure	36 % (42)	47 % (55)
Peripheral vascular disease	8 % (10)	13 % (15)
Cerebrovascular disease	39 % (46)	23 % (27)
Dementia	2 % (2)	2 % (2)
Chronic pulmonary disease	16 % (19)	12 % (14)
Connective tissue disorder	3 % (4)	9 % (11)
Ulcer disease	8 % (10)	7 % (11)
Mild liver disease	2 % (2)	3 % (3)
Diabetes mellitus without complications	47 % (55)	50 % (59)
Diabetes mellitus with end-organ complications	43 % (51)	41 % (48)
Hemiplegia	5 % (6)	3 % (3)
Tumor without metastases	16 % (19)	14 % (17)
Leukemia, lymphoma, multiple myeloma	1 % (1)	1 % (1)
Moderate or severe liver disease	2 % (2)	1 % (1)
Metastatic solid tumor	0 % (0)	1 % (1)
AIDS	0 % (0)	0 % (0)
